# Inequality in Diabetes-Related Hospital Admissions in England by Socioeconomic Deprivation and Ethnicity: Facility-Based Cross-Sectional Analysis

**DOI:** 10.1371/journal.pone.0116689

**Published:** 2015-02-23

**Authors:** Yoshitaka Nishino, Stuart Gilmour, Kenji Shibuya

**Affiliations:** Department of Global Health Policy, Graduate School of Medicine, University of Tokyo, 7-3-1 Hongo, Bunkyo-ku, Tokyo, 113-0033, Japan

## Abstract

**Objective:**

To investigate the effect of social deprivation and ethnicity on inpatient admissions due to diabetes in England.

**Design:**

Facility-based cross-sectional analysis.

**Setting:**

National Health Service (NHS) trusts in England reporting inpatient admissions with better than 80% data reporting quality from 2010–2011 (355 facilities).

**Participants:**

Non-obstetric patients over 16 years old in all NHS facilities in England. The sample size after exclusions was 5,147,859 all-cause admissions.

**Main Outcome Measures:**

The relative risk of inpatient admissions and readmissions due to diabetes adjusted for confounders.

**Results:**

There were 445,504 diabetes-related hospital admissions in England in 2010, giving a directly (age-sex) standardized rate of 1049.0 per 100,000 population (95% confidence interval (CI): 1046.0–1052.1). The relative risk of inpatient admission in the most deprived quintile was 2.08 times higher than that of the least deprived quintile (95% CI: 2.02–2.14), and the effect of deprivation varied across ethnicities. About 30.1% of patients admitted due to diabetes were readmitted at least once due to diabetes. South Asians showed 2.62 times (95% CI: 2.51 – 2.74) higher admission risk. Readmission risk increased with IMD among white British but not other ethnicities. South Asians showed slightly lower risk of readmission than white British (0.86, 95% CI: 0.80 – 0.94).

**Conclusions:**

More deprived areas had higher rates of inpatient admissions and readmissions due to diabetes. South Asian British showed higher admission risk and lower readmission risk than white British. However, there was almost no difference by ethnicity in readmission due to diabetes. Higher rates of admission among deprived people may not necessarily reflect higher prevalence, but higher admission rates in south Asian British may be explained by their higher prevalence because their lower readmission risk suggests no inequality in primary care to prevent readmission. Better interventions in poorer areas, are needed to reduce these inequalities.

## Introduction

It is well known that socioeconomic deprivation is associated with excess hospital admissions. For example, people in unskilled employment[1], people from a household whose head is in an unskilled-labour social class, or with an adult member of the family who cannot work due to illness, experience higher rates of emergency admission[2]. In the United States, people in highly deprived socioeconomic conditions or from certain ethnic minorities have a higher risk of diabetes-related hospitalization[3]. To the extent that these inequalities in admission rates are preventable, they represent a burden of potentially avoidable hospital admissions which, if addressed, may lead to health service efficiencies.

Diabetes is a typical example of a chronic non-communicable disease. Diabetes patients need continuous careful management in primary care by a general practitioner (GP) to prevent complications that may lead to hospitalization. In Type 1 diabetes, failure of primary care management can lead to acute ketoacidosis, which requires immediate emergency department admission; acute sequelae of untreated or poorly-managed type 2 diabetes can also lead to preventable hospital admissions. In the context of a growing diabetes epidemic in the UK, prevalence of type 1 and type 2 diabetes are expected to increase from approximately 0.6% and 5.4% of the whole population respectively in 2010/2011 to 0.9% and 7.7%, respectively by 2035/2036[4], for assumed population projections at 2035[5]. The annual cost of emergency calls for severe hypoglycaemia is £13.6m for England alone[6], and the total cost of diabetes is estimated to increase from £23.7bn in 2010/2011 to £39.8bn by 2035/2036[4]. Reducing excess inpatient admissions, especially in high-prevalence groups, is important to both reduce unnecessary expenditure on medical services and to reduce demand on NHS hospitals.

In the UK the prevalence of diabetes is estimated to be almost equal across socioeconomic status in men and at most double in women between the highest and lowest social classes[7]. Thus, a socioeconomic gradient in excess hospital admissions due to diabetes in the UK may reflect poorer management of chronic diabetes in this group. Some research also suggests inequality in diabetes primary care by ethnicity[8]. British of black Caribbean or south Asian descent are known to have a higher prevalence of diabetes[9–11] and without good quality management in primary care it is likely that this higher prevalence will be represented in higher rates of emergency admissions.

Excess rates of admission for diabetes represent a health system burden that can be reduced through targeting better quality primary care management of diabetes[12] such as controlling blood glucose concentration and blood pressure[13]. In a period of straitened finances in the UK and significant cuts to both social welfare and health services[14], it is important to understand the role that socioeconomic inequality can play in increasing the burden on health systems, to better prepare for and manage its health system effects. Furthermore, the current agenda for NHS reform in the UK focuses on increasing the role of GPs in clinical commissioning and health service planning[15], suggesting that GPs will be playing a greater future role in public health than has previously been the case, with the risk that flaws in primary care service will have a greater impact on the health system in the future. In this study, we investigate the relationship between socioeconomic deprivation, ethnicity and inpatient admission for diabetes using data for all of England.

## Methods

### Data sources

Data on individual inpatient hospital admissions was obtained from the inpatient components of the UK Hospital Episode Statistics (HES) data set. This data contains individual records of all attendances occurring in England from 2010–11, and was obtained from the National Health Service (NHS) Information Centre[16]. The population data for Lower Super Output Areas (LSOAs), the key geographical variable on which data on socioeconomic deprivation is available for 2011, was obtained from the website of the Office for National Statistics[17]. This data set contained 8,696,242 all-cause admissions in 498 hospitals.

Patients with obstetric-related diagnostic codes were excluded because gestational diabetes has very different etiology, management and epidemiological patterns than adult diabetes. In the inpatient data, only patients admitted through elective and emergency admission pathways were analyzed and other admission methods (maternity, psychiatric etc.) were excluded.

To avoid duplication of patient data due to follow-up admission, between- or within-facility referral for the same admission period, only the initial admission record for any hospital stay (the first episode of a “spell” in HES terminology) was analyzed This exclusion criterion is standard practice for analysis of admission data in the inpatient dataset[16]. Patients under 16 years old were also excluded from this analysis. Admissions from postcodes in Wales, Scotland, Northern Ireland, the Channel Islands, the Isle of Man, and any “pseudopostcodes” were excluded because these postcodes lack information about the Index of Multiple Deprivation (IMD), and only data for England were analyzed.

### Missing data

Before analysis, records were excluded on a facility-wise basis depending on the rate of missing data in key variables. Hospitals with less than 80% valid data on diagnosis were excluded from the data set. Because ethnicity is relatively poorly recorded in the data, analysis of the relationship between hospital admission and ethnicity was restricted to only those hospitals with at least 80% valid data on both diagnosis and ethnicity.

### Age-sex standardization of emergency admission rate

Directly standardized inpatient admission rates per 100,000 population were calculated as the age- and sex-standardized mean of the admission rates of each IMD quintile using the whole of England as the reference population. All statistical analysis was conducted on crude admission counts in order to adjust for age and sex.

### Covariates

Admission due to diabetes was defined as a hospital admission with primary or secondary diagnosis from the ICD 10 codes E10—E14. IMD was used to express the degree of socioeconomic deprivation. This index is given at the level of small districts called Lower Super Output Areas, each consisting of about 672 households on average[18], and aggregates indices measuring income, employment, health, education, housing services, crime, and the living environment[19]. We adjusted all analyses for the confounding effect of patients’ sex, ethnicity, age on the day of admission, and rural-urban indicator. In the HES data, rural-urban indicator is classified into nine categories[20] representing degree of urbanization. For this analysis, we collapsed them into three: urban, town, and village/hamlet. The admission method, elective or emergency, was also adjusted for.

HES data classifies ethnicity into 18 categories[20], but we grouped data into six categories: white, mixed, south Asian, black, Asian, and others.

### Statistical analysis

The relationship between regional deprivation and inpatient admission was analyzed using a multiple Poisson regression model with random effects for region (the LSOA) and IMD treated as a region-level variable. Population by broad age groups (16–29, 30–44, 45–64, over 65) at the LSOA level was used as an offset in the regression model. All results from the multiple regression models are presented as relative risks with confidence intervals and p-values. We categorized IMD into quintiles and age into the same broad age groups as were present in the population data. An interaction term between IMD and ethnicity was included in the model to identify effect modification due to ethnicity.

### Analysis of high-impact users

Using the same set of inpatient admission data, patients readmitted to hospital for diabetes were identified and their total number of readmissions calculated. To adjust for varying dates of initial admission, the time at risk was calculated in person-years from the first admission to the end of the data collection period (31^st^ March 2011). A small number of patients whose record of observation period exceeded one year were excluded (20,352 patients). After exclusion, 230,535 patients remained. The number of readmissions was analyzed using multi-level Poisson regression. However, a small number of extreme outliers (more than 11 readmissions: 0.05% of the whole dataset) were excluded due to computational problems. The natural log of the person-year was used as the offset in the Poisson regression, and LSOA-level population was excluded because the analysis was only being conducted on the subset of those admitted from each area. Admission method of the initial admission due to diabetes for each patient was included as a covariate in this model, to test the relative risk of readmission of emergency compared to elective patients.

### Ethics Considerations

Approval was given for the use of the data by the NHS Information Centre. Because the data is anonymous, routine monitoring data open to the public, further ethics approval is not required.

## Results

**Fig 1:**
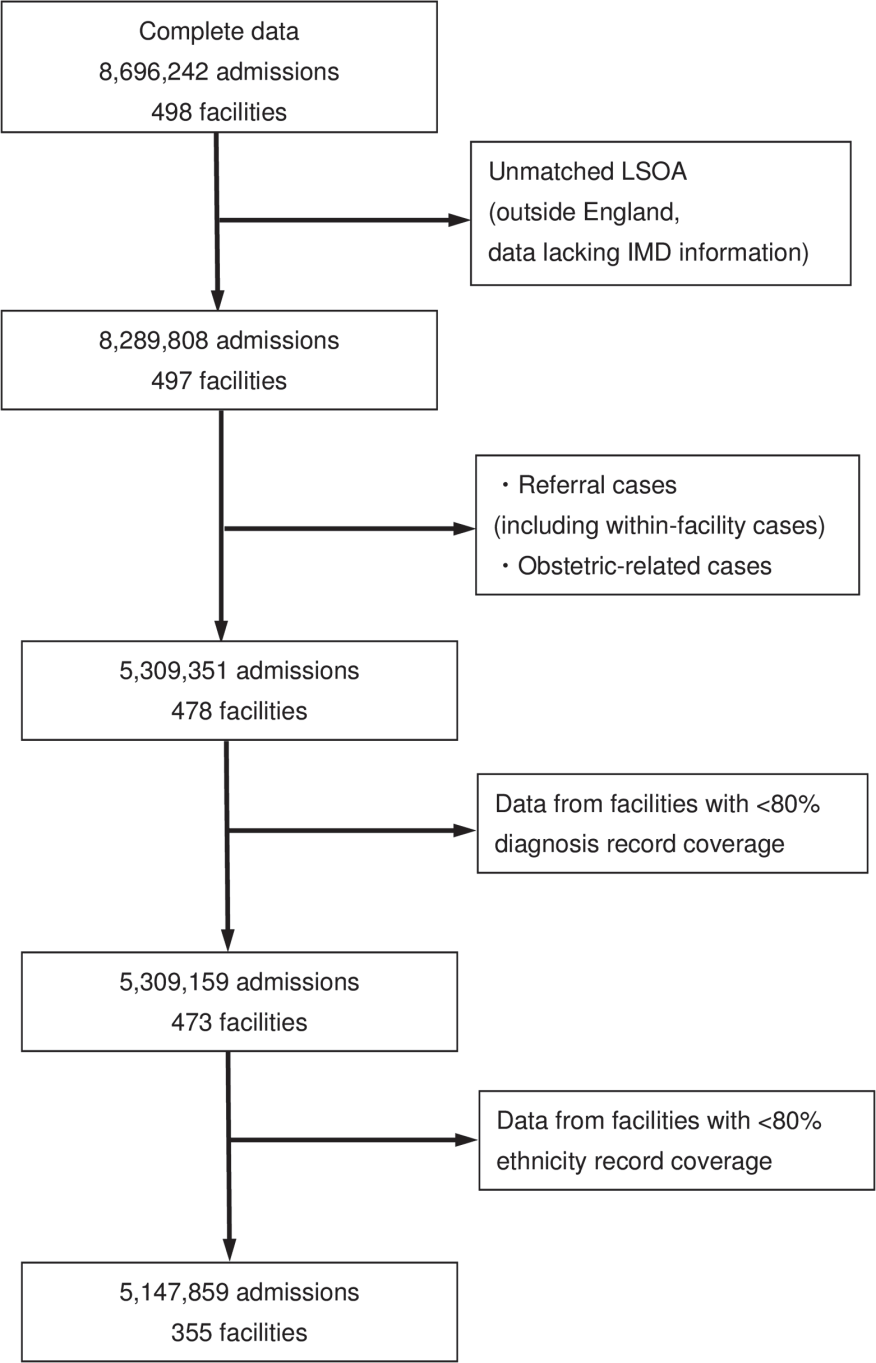
Data exclusion flowchart.

A flowchart describing the application of exclusion criteria and removal of missing data is summarized in Fig. 1. There were a total of 8,696,242 all-cause admissions in 498 hospitals. After applying the exclusion criteria, 5,309,351 admissions remained, spread across 478 hospitals. After excluding data with insufficient diagnostic codes and ethnicity data, 5,147,859 records remained in the data set, spread over 355 NHS hospitals. This is 97.0% of all valid attendances and 74.3% of all valid NHS hospitals.

In the whole of England, there were a total of 445,504 diabetes admissions, giving a crude rate of 1049.0 (95% CI: 1046.0–1052.1) admissions per 100,000 population.

**Fig 2:**
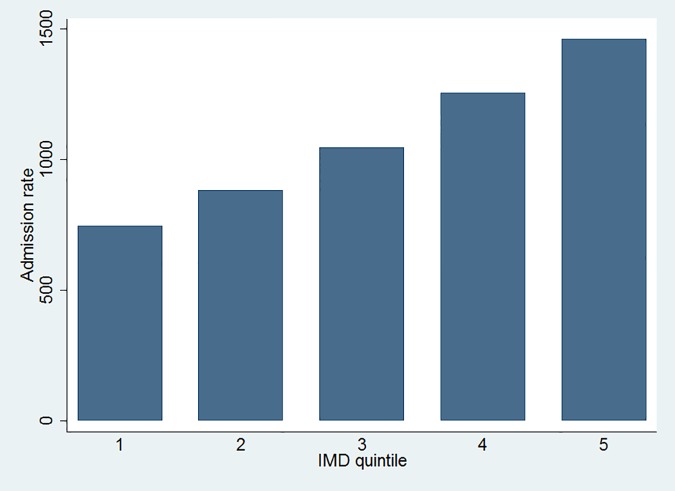
IMD quintile-specific directly standardized admission rate due to diabetes.

Fig. 2 shows IMD quintile-specific directly standardized admission rates. The age-sex standardized admission rate increases with increasing deprivation.

Table 1 shows the result of multiple regression analysis of diabetes admissions by IMD quintile adjusting for ethnicity. Likelihood ratio tests comparing the multi-level model with a simple Poisson regression without random effects in the model showed the random effects were significant. The relative risk of diabetes-related admission increases with IMD quintile. The poorest quintile of white British had a 2.08 times (95% CI 2.02–2.14) higher admission risk than the richest quintile. The risk of diabetes-related admission among south Asian British was 2.62 (95% CI 2.51–2.74) times higher than white British. Although statistically significant, the risk increase among other non-white British was not large. Other risk factors for hospital admission were age, sex and living in an urban area.

**Table 1:** Multiple regression model of relationship between hospital admission due to diabetes, IMD quintile and ethnicity.

**Variables**	Relative Risk	95% Confidence Interval	P-value
**IMD quintile**			
Quintile 1 (Least Deprived)	1	N.A.	
Quintile 2	1.16	1.13–1.19	<0.001
Quintile 3	1.35	1.32–1.39	<0.001
Quintile 4	1.68	1.63–1.73	<0.001
Quintile 5 (Most Deprived)	2.08	2.02–2.14	<0.001
**Age**			
16–29 years	1	N.A.	
30–44 years	2.28	2.22–2.34	<0.001
45–64 years	5.88	5.74–6.02	<0.001
65 + years	14.17	13.83–14.51	<0.001
**Sex**			
Men	1	N.A.	
Women	0.64	0.63–0.64	<0.001
**Region type**			
Urban	1	N.A.	
Town	0.89	0.87–0.91	<0.001
Village	0.81	0.80–0.83	<0.001
**Ethnicity**			
White	1	N.A.	
Mixed	1.49	1.29–1.72	<0.001
South Asian	2.62	2.51–2.74	<0.001
Black	1.22	1.09–1.37	0.001
Asian	1.02	0.87–1.21	0.783
Others	1.32	1.21–1.43	<0.001
**IMD/ethnicity interaction**			
IMD Quintile 1: all	1	N.A.	
IMD Quintile 2:			
Mixed	0.83	0.68–1.01	0.061
South Asian	0.91	0.86–0.97	0.004
Black	1.23	1.07–1.42	0.004
Asian	1.22	0.95–1.57	0.111
Others	1.03	0.92–1.16	0.585
IMD Quintile 3:			
Mixed	1.15	0.97–1.37	0.10
South Asian	0.85	0.81–0.91	<0.001
Black	1.20	1.05–1.36	0.006
Asian	0.83	0.65–1.06	0.135
Others	1	0.90–1.11	0.946
IMD Quintile 4:			
Mixed	0.73	0.61–0.86	<0.001
South Asian	0.81	0.77–0.86	<0.001
Black	1.12	0.99–1.26	0.068
Asian	1.10	0.89–1.36	0.371
Others	0.89	0.80–0.98	0.019
IMD Quintile 5:			
Mixed	0.86	0.73–1.01	0.074
South Asian	0.81	0.77–0.85	<0.001
Black	1.10	0.97–1.24	0.127
Asian	1.07	0.87–1.32	0.538
Others	0.86	0.78–0.95	0.003
**Admission method**			
Elective	1	N.A.	
Emergency	1.61	1.60–1.62	<0.001
**Likelihood ratio statistic against without random effect mode (p-value)**	129728.94(<0.001)		

**Fig 3:**
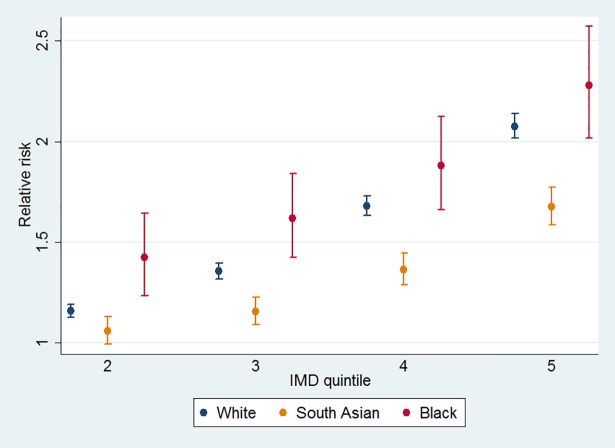
Sensitivity of relative risk of admission to IMD quintiles by ethnicity.

The chi square value of the Wald likelihood test of overall interaction terms (185.25, p-value <0.001) shows that there was a statistically significant interaction between ethnicity and deprivation. Fig. 3 shows the relationship between diabetes-related hospital admission risk and IMD quintile, for selected ethnicities calculated from linear combinations of the coefficients shown in Table 1. The effect modifier for south Asian ethnicity showed lower sensitivity to IMD (1.68 times higher risk in the most deprived quintile than the least deprived quintile) than black British (2.28 times higher risk) or white British (2.08 times higher risk). This is suggestive of an attenuated effect of deprivation in south Asian British relative to white and black British.

A total of 174,932 patients were admitted due to diabetes at least once and 73,684 patients were admitted more than once. Table 2 shows the result of Poisson regression of readmission in these patients. For this model the random effect was also significant. The relative risk of diabetes-related readmission also increases with IMD quintile but the association was much weaker than that of admission. The poorest quintile of white British had a 1.18 times (95% CI 1.15–1.22) higher readmission risk than the richest quintile. The relative risk of readmission in south Asian British was 0.86 (95% CI 0.80–0.94) times that of white British. Other ethnic groups did not show significant difference from white British except the “other” ethnic group. Readmission risk was slightly higher in patients whose index admission was elective than those who were admitted through the emergency department. Women showed slightly lower risk of readmission than men. Almost all other covariates showed no significant difference.

**Table 2:** Multiple regression model of relationship between number of hospital readmissions due to diabetes, IMD quintile and ethnicity.

**Variables**	Relative Risk	95% Confidence Interval	P-value
**IMD quintile**			
Quintile 1 (Least Deprived)	1	N.A.	
Quintile 2	1.02	0.99–1.05	0.166
Quintile 3	1.06	1.03–1.10	<0.001
Quintile 4	1.13	1.09–1.16	<0.001
Quintile 5 (Most Deprived)	1.18	1.15–1.22	<0.001
**Age**			
16–29 years	1	N.A.	
30–44 years	0.91	0.87–0.96	<0.001
45–64 years	0.97	0.93–1.01	0.128
65 + years	1.02	0.97–1.06	0.453
**Sex**			
Men	1	N.A.	
Women	0.90	0.89–0.91	<0.001
**Region type**			
Urban	1	N.A.	
Town	1.03	1.00–1.06	0.079
Village	1.01	0.98–1.03	0.723
**Ethnicity**			
White	1	N.A.	
Mixed	1.01	0.79–1.29	0.964
South Asian	0.86	0.80–0.94	0.001
Black	1.03	0.84–1.26	0.779
Asian	0.73	0.53–1.01	0.058
Others	1.16	1.02–1.33	0.026
**IMD ethnicity interaction**			
IMD Quintile 1: all	1	N.A.	
IMD Quintile 2:			
Mixed	1	0.71–1.39	0.981
South Asian	1.06	0.95–1.19	0.270
Black	1.11	0.87–1.42	0.383
Asian	0.96	0.59–1.57	0.111
Others	0.77	0.63–0.93	0.007
IMD Quintile 3:			
Mixed	1.17	0.86–1.58	0.313
South Asian	1.04	0.94–1.14	0.499
Black	0.92	0.73–1.14	0.432
Asian	0.80	0.49–1.32	0.387
Others	0.77	0.65–0.92	0.003
IMD Quintile 4:			
Mixed	0.92	0.69–1.23	0.582
South Asian	1.03	0.94–1.14	0.495
Black	0.92	0.74–1.13	0.411
Asian	1.29	0.85–1.95	0.230
Others	0.91	0.78–1.07	0.264
IMD Quintile 5:			
Mixed	0.78	0.58–1.04	0.092
South Asian	0.97	0.88–1.07	0.543
Black	0.92	0.74–1.13	0.404
Asian	1.80	1.22–2.65	0.003
Others	0.82	0.70–0.96	0.014
**Admission method**			
Elective	1	N.A.	
Emergency	0.85	0.84–0.86	<0.001
**Likelihood ratio statistic against without random effect model (p-value)**	9465.53 (<0.001)		

**Fig 4:**
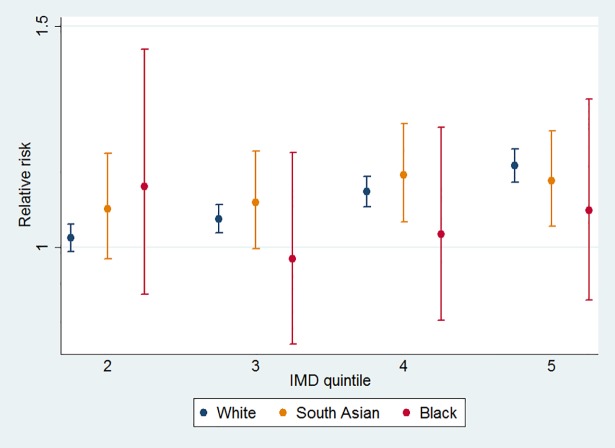
Sensitivity of relative risk of readmission to IMD by ethnicity.

Fig. 4 shows the relationship between readmission and IMD by selected ethnicities, calculated from linear combinations of the coefficients shown in the Poisson regression. Black and white British show almost no difference by IMD quintiles, while hospital readmission in more deprived groups was higher in white British though the relationship is attenuated.

## Discussion

This is the first study to analyze the effect of socioeconomic deprivation and ethnicity on inpatient hospital admission and readmission due to diabetes using inpatient admission data for all of England, by deprivation quintiles and by ethnic groups. Our study is the first analysis of HES data for the whole of England to use multi-level modeling in the statistical analysis. We used IMD as a second level effect, because IMD is calculated at the level of LSOA rather than for individuals[19]. This enables our study to incorporate the effect of unmeasured community-level influences on hospital admission risk.

In this study we found that risk of inpatient admission for diabetes increases with increasing socioeconomic deprivation measured by IMD quintile, and that although the relationship exists across all ethnicities it is strongest in white and black British. The relative risk of hospital admission in the most deprived quintile among white British was 2.08 (95% CI 2.02–2.14) times higher than the least deprived quintile. The relative risk in the most deprived quintile was 1.68 (95% CI: 1.58–1.77) times and 2.28 (95% CI: 2.02–2.57) times higher than the least deprived quintile among south Asian and black British, respectively. Admission risk was 1.61 times (95% CI: 1.60–1.62) higher for transfer from emergency departments than through elective or planned admission pathways.

This research also found that ethnic minority groups are at higher risk of hospital admission for diabetes compared to white British people even after adjusting for socioeconomic deprivation. For diabetes, this may reflect the known differences in prevalence between South Asian, white and black British[9–11]. We found readmission rates in most non-white groups were not significantly different from the white group, though the smaller number of observations gave wider confidence intervals. This suggests that the relationship between ethnicity and inpatient admission may reflect racially-specific higher prevalence, but may also be a function of differences in primary care management or health-seeking behavior[21]. By contrast, this study found that people from poorer areas had higher rates of readmission, even after adjusting for ethnicity. Even though they were admitted once, after discharge the same people in these socioeconomic groups were more likely to be admitted to hospital again. This suggests that prevalence alone does not account for the differences in admission risk by IMD.

The two main possible causes of the increased risk of hospital admissions and readmissions in poorer areas are excess prevalence of individual risk factors in poorer individuals, or poorer management of diabetes in primary care in poorer areas[22]. One of the most important risk factors for diabetes is obesity, and the prevalence of obesity is inversely associated with socioeconomic status in developed nations[23]. Type 2 diabetes prevalence increased in the UK between 1994 and 2006 and the increase was associated with socioeconomic inequality measured by social class categories[7], though there was no consistent pattern in individual risk factors[24].

In the UK, the poor and ethnic minorities are more likely to consult their GP than secondary care[25] and people from more deprived socioeconomic backgrounds use all forms of medical services less frequently[8]. Although historical socioeconomic disparities in diabetes care[26] seem to have reduced[24], it has been suggested that there is still poorer control of risk factors such as HbA_1c_ in more deprived white British[27]. In the context of the pathway of care through Britain’s health system, this result suggests a failure of the primary care gate-keeping role, leading to increased need for secondary care for diabetic patients in highly deprived areas. In the most deprived areas and the areas with the worst health and deprivation indicators (so-called Spearhead areas), GPs obtain lower scores on the quality and outcomes framework (QOF), an indicator of quality of primary care. Practices in Spearhead areas have lower numbers of GPs per practice and higher caseloads for each GP, which may lead to lower quality of practice[28]. Poorer management of diabetes and higher prevalence of undiagnosed diabetes, combined with restricted access to medical services and poorer patterns of health-seeking behavior, is likely to lead to an increased burden on secondary care services, and our results confirm this increased service use.

Higher rates of admission we observed in some ethnicities may also be partially explained by higher prevalence. In a recent study, the age-standardized prevalence of diabetes in black British and south Asian descendants was two or three times that of white British, respectively[29]. The higher risk of inpatient admission is consistent with this difference in prevalence. There is some evidence that black and south Asian British are treated with HbA_1c_ control more intensively[30], although findings about quality of primary care in ethnic minorities are inconsistent[8,31]. We could not adjust for body mass index (BMI) in this analysis due to unavailability of this data in the HES data, and the high rates of admission in some ethnicities may be partly explained by racial differences in the role of this risk factor. A Canadian study has shown that south Asian and black people can develop diabetes at lower BMI thresholds than white people, though the mechanism is not understood[32]. Recent NICE guidance on assessing BMI thresholds finds that south Asian and black people may have equivalent risk in lower BMI than white people, but the guidance does not specify ethnicity-specific thresholds due to insufficient evidence[33]. The result of our study suggests that primary care in these ethnic minorities may not be sufficiently managed, though the guidance recommends multi-component interventions in ethnic minorities[33]. On the other hand, in light of this potential differential risk structure, the finding of almost no difference in readmission rates among non-white ethnicities and slightly lower risk of readmission in south Asians may mean this more intensive care in these ethnic groups may have improved diabetic condition and reduced the risk of readmission. Considering these results, first diagnosis of diabetes may be a key to address the inequality in risk of admissions. A better understanding of both prevalence and readmission pathways is necessary in these ethnicities to better understand the causes of the inequalities observed in this study.

Because this study uses a secondary data collection, it is subject to several limitations. We could not adjust for any individual confounding factors beyond age and sex, so could not adjust for the confounding effect of health background including severity of disease, occupation, employment status, educational history or whether patients have already received medical care against diabetes before hospital admission. Future research using data linking hospital admissions and primary care databases should focus on the pathway from primary to secondary care, to identify possible causes of the increased risk of diabetes-related admission in more socio-economically deprived areas.

This study has found a strong association between socioeconomic deprivation and ethnicity and diabetes-related inpatient admission. Action needs to be urgently taken to reduce preventable hospital admissions by:

- Increasing attention on the quality of management of NCDs and their risk factors in primary care, especially in “Spearhead” areas with large deprived populations. Current attempts to improve primary care management of diabetes are focused on the quality and outcomes framework (QOF) but there is limited evidence that this is working effectively in “Spearhead” areas. Furthermore, south Asian and Black patients are more likely to be excluded from QOF measures, and these excluded patients are less likely to achieve intermediate treatment targets[34]. To avoid such problems, the incentive structure built into this program needs to be changed to better reflect the growing NCD epidemic
- Increasing community awareness of diabetes and promoting healthy behavior through mass media or education emphasizing the importance of early diagnosis, better dietary and blood-sugar monitoring amongst those already diagnosed, and more regular primary care visits. These screening, prevention and management efforts should be especially focused in highly deprived areas
- Development of innovative methods to target deprived areas and high-risk ethnic groups for improvements in diabetes treatment and management. This will include improvements in community nursing and continuity of care; development of care plans implementable in primary care and easily understood by patients; inclusion in care plans of targets to reduce inpatient admissions; and improvements in timeliness of access to outpatient care. These methods will need to use the new coordination and funding powers of the clinical commissioning groups (CCGs) that were established in April 2013[35].
- Constructing a nationally standardized referral database which can be shared by all NHS facilities, CCGs and GPs to understand and improve referral patterns. This will require renewed commitment to information systems reform, which will be challenging given resource constraints and the limited progress of the NHS Care Records Service[36].

The UK diabetes research agenda needs to consider ways to improve research on the relationship between diabetes prevalence and socioeconomic deprivation and ethnicity. Better, more accurate measures of prevalence of diabetes are essential in order to better understand the extent of the problem in England, and to enable measures of hospital admission intensity to be standardized by disease prevalence rather than total population. These measures can be achieved by expanding the coverage and scope of the Health Survey for England, over-sampling areas with high ethnicities and deprived areas, and increasing the detail and scope of the NCD sections of this survey, and also by using the central coordinating authority of the Information Centre to identify local and regional datasets that may contain important information on regional and local variations in prevalence. Improvements in collection of ethnicity and diagnostic codes in the hospital episode statistics data are also essential.

Inequality in health outcomes as a result of NCDs is not inevitable and can be reversed. For example, IMD has no influence on survival due to heart failure and numbers of patients with a first hospital admission with heart failure reached a plateau in 1998[37]. This suggests that medical care for heart failure has been improved for people from all socioeconomic backgrounds, but for diabetes is still insufficient. Through better attention to primary care management of diabetes, at least some part of the inequity in health outcomes experienced by the most deprived members of British society can be reduced.
